# Artificial outdoor light at night and depression in older adults in the USA, England, Northern Ireland, and Ireland

**DOI:** 10.1016/j.envint.2025.109886

**Published:** 2025-10-27

**Authors:** Rina So, Jennifer D’Souza, Joanne Feeney, Hüseyin Küçükali, Kayleigh P. Keller, Giorgio Di Gessa, Joanna Sara Valson, Ruth F. Hunter, Bernadette McGuinness, Frank Kee, Anne Nolan, Sinead Mc Loughlin, Jinkook Lee, Sara D. Adar, Paola Zaninotto

**Affiliations:** a Department of Epidemiology and Public Health, University College London, London, United Kingdom; b Section of Environmental Health, Department of Public Health, University of Copenhagen, Copenhagen, Denmark; c Department of Epidemiology, The University of Michigan, Ann Arbor, MI, USA; d The Irish Longitudinal Study on Ageing (TILDA), Trinity College, Dublin, Ireland; e Centre for Public Health, Queen’s University Belfast, Belfast, United Kingdom; f Research Center for Healthcare Systems and Policies, İstanbul Medipol University, İstanbul, Türkiye; g Department of Statistics, Colorado State University, Fort Collins, CO, USA; h Department of Economics, Trinity College Dublin, Dublin, Ireland; i Economic and Social Research Institute, Dublin, Ireland; j Department of Economics & Center for Economic and Social Research, University of Southern California, CA, USA; k Department of Epidemiology, University of Michigan, MI, USA

**Keywords:** Aging, Environment exposure, Light at night, Light pollution, Longitudinal

## Abstract

**Background:**

Artificial Light at night (ALAN) is a potential environmental stressor for depression, but epidemiological evidence is limited. Cross-national surveys of aging were leveraged to examine LAN and depression.

**Methods:**

We used longitudinal aging surveys from the US (HRS; n = 20,868), England (ELSA; n = 9848), Ireland (TILDA; n = 6407), and Northern Ireland (NICOLA; n = 2725). Depression was ascertained using Center for Epidemiologic Studies Depression Scale and dichotomized based on study-specific cutoffs. Annual mean outdoor ALAN exposure was estimated using satellite-derived nighttime light data (~500 m resolution), then categorized using harmonized quartiles, based on cross-country population values (≤2.79, 2.79-11.69, 11.69-23.93 and *>*23.93 nW/cm^2^/s). Poisson regression models estimated the prevalence ratios (PRs) of depression, adjusting for individual- and area-level factors.

**Results:**

The prevalence of depression was highest in ELSA and HRS (24%), followed by NICOLA (14%) and lowest in TILDA (8%). The mean (SD) ALAN levels were 18.9 (9.0) nW/cm^2^/s in HRS, 13.4 (12.7) in ELSA, 10.4 (13.4) in TILDA, and 11.6 (10.3) in NICOLA. In fully-adjusted models, the highest ALAN quartile was associated with higher PRs of depression (reference: lowest quartile), in all surveys, with PRs (95% Confidence interval) of 1.40 (1.20–1.63) in HRS, 1.16 (0.98–1.38) in ELSA, 1.51 (1.08–2.10) in TILDA, and 1.79 (1.13–2.84) in NICOLA. The directions of the association were robust to adjustment for NO2, though attenuated.

**Conclusions:**

Findings from multiple countries suggest that outdoor ALAN exposure is associated with depression in older adults and highlight the value of international longitudinal aging cohorts for investigating the impact of environmental exposures on health.

## 1. Introduction

Exposure to outdoor artificial light at night (ALAN) is a common feature of modern life, largely due to urbanization, which includes streetlights, exterior and interior building lighting, as well as industrial and commercial lighting. (Zielińska-Dabkowska et al., 2020) While artificial lighting is a significant technological advancement that enhances human activity and productivity at night, and increases perceptions of safety, excessive exposure to LAN has been reported to disrupt natural circadian rhythm. (Fonken and Nelson, 2014) Circadian disruption has been linked with various health conditions, such as metabolic (Fonken and Nelson, 2014; Nelson and Chbeir, 2018), cardiovascular (Münzel et al., 2021; Chellappa et al., 2019) diseases, breast cancer, (Luo et al., 2023) and depression. (Walker et al., 2020).

Depression is a leading source of disability worldwide (Ferrari et al., 2024) and is associated with reduced life quality, particularly in older populations, (Noel, 2004) as well as an increased risk of morbidity and mortality. (Schulz et al., 2000; Padayachey et al., 2017) Various risk factors for depression have been identified, especially in older adults, including sleep disturbances, hearing problems, poor vision, cardiac diseases, (Wu et al., 2022) and air pollution. (Borroni et al., 2022) Recently, the psychological impact of exposure to ALAN has emerged as a concern, (Tancredi et al., 2022) with suggested biological pathways including sleep disturbance and circadian disruption, as observed in animal studies. (An et al., 2020; Tam et al., 2021) Older adults may be particularly vulnerable to the effects of ALAN due to age-related changes, such as disrupted sleep patterns, reduced melatonin production, and a higher prevalence of chronic conditions, which can weaken circadian regulation. (Mattis and Sehgal, 2016; Lananna and Musiek, 2020).

A recent systematic review of nine studies reported moderate evidence of a positive association between ALAN exposure and depressive symptoms. (Tancredi et al., 2022) However, only a few studies specifically examined older adults, (Obayashi et al., 2013; Obayashi et al., 2018; Wallace-Guy et al., 2002) and findings in these populations were inconsistent. Additionally, the review highlighted several limitations, including the small number of studies and the potential influence of confounding factors, such as socio-economic status, noise, or air pollution. (Tancredi et al., 2022) Furthermore, there is a lack of standardized ALAN exposure assessments and comparable depression outcome measures across studies. Notably, ALAN exposure and its effects may vary between urban and rural settings due to differences in light sources, environmental exposures, and adaptation behaviors, yet these geographic differences in relation to mental health outcomes remain understudied.

In this study, we aim to examine whether long-term exposure to outdoor ALAN is associated with depressive symptoms in older populations using harmonized exposome data from four longitudinal studies of aging in high income settings of North America and Europe that are part of the Gateway to Global Aging Data project (Gateway) and the Health and Retirement Study (HRS) International Network of Studies.

## 2. Materials and methods

### 2.1. Study population

We used data from four nationally representative aging studies of adults aged 50 years or older in the U.S. (HRS), (Sonnega et al., 2014) England (the English Longitudinal Study of Ageing, ELSA), (Steptoe et al., 2013) Ireland (The Irish Longitudinal Study of Ageing, TILDA), (Donoghue et al., 2018) and Northern Ireland (Northern Ireland Cohort for the Longitudinal Study of Ageing; NICOLA). (Neville et al., 2023) These are nationally-representative longitudinal cohorts of older adults designed to understand risk factors for healthy aging in different populations. Full details of survey design and cohort characteristics are provided elsewhere; (Sonnega et al., 2014; Steptoe et al., 2013; Donoghue et al., 2018; Neville et al., 2023) and we provide a description of each study in the Supplementary Text S1.

Based on availability of the ALAN and survey data, for this study we used waves 11–13 of HRS (2-years periods in 2012–2016), waves 6–9 of ELSA (surveyed every 2 years from 2012 to 2019), waves 2–5 of TILDA (surveyed every two years from 2012 to 2018), and wave 1 (December 2013-July 2016) of NICOLA.

We included individuals at each wave with complete responses for all questions on depressive symptoms and on covariates. If a participant had missing covariates in certain waves, their data were excluded only from those specific waves but retained for other waves where complete information was available. Participants gave their consent to participate in each study.

HRS protocols are reviewed and approved by the Health Sciences and Behavioral Sciences Institutional Review Board at the University of Michigan, and all participants provide informed consent prior to participation, in line with ethical research standards. Ethical approval for ELSA was obtained from the NRES Committee South Central – Berkshire, and informed consent was obtained from all individual participants included in the study. For TILDA, ethical approval was obtained from the Trinity College Dublin Faculty of Health Sciences Research Ethics Committee, and written informed consent was provided by all participants, in line with the Declaration of Helsinki. Ethical approval for the NICOLA was obtained from the School of Medicine, Dentistry and Biomedical Sciences Ethics Committee, Queen’s University Belfast, and written informed consent for the use of NICOLA data collected via questionnaires and during the clinical based health assessment was obtained from participants following the recommendations of the School of Medicine, Dentistry and Biomedical Sciences Ethics Committee, Queen’s University Belfast at the time. Please note that the NICOLA study website contains details of all the data that is available through a fully searchable data dictionary. (Queen’s University Belfast, 2025).

### 2.2. Measure of depressive symptoms

Depressive symptoms in the week prior to the interview were measured using the Centre for Epidemiological Studies Depression (CES-D) scale in all surveys. This scale has been previously validated against gold-standard psychiatric interviews, with good sensitivity and specificity. (Radloff, 1977) The version and the number of items varies across the four surveys and waves. In HRS and ELSA, a short version of the CES-D was used, consisting of 8 items with binary response options (yes or no; scored 1 or 0, respectively). (Turvey et al., 1999) This abridged version has good internal consistency at each wave (Cronbach’s α > 0.95) and comparable psychometric properties to the full 20-item CES-D. (Turvey et al., 1999) In TILDA, the original 20-item CES-D, which uses a 4-point scale (score ranging from 0 to 3), was administered in wave 2. In subsequent waves, the 8-item version was used, similar to the one in ELSA and HRS, but with a 4-point scale answer. In NICOLA, the original 20-item CES-D, with a 4-point scale response, was used. A list of questionnaire items for these different versions is provided in Supplementary Table S1. For TILDA, where different versions were used before and after wave 2, we selected the 8 items from the original version in wave 2 that overlapped with the short version to ensure comparability across waves. A previous study validated this 8-item version against the full 20-item version in TILDA and found it to be a valid and reliable measure of depressive symptoms in this sample. (Briggs et al., 2018) We summed all items in the scales, with maximum scores of 8 in ELSA and HRS, 24 for TILDA, and 60 for NICOLA. To derive a clinically meaningful cut-off for the presence of depressive symptoms and ensure comparability across studies, depressive symptoms were dichotomized based on study-specific and previously validated thresholds: ≥4 in ELSA and HRS, (Steffick, 2000) ≥ 9 for TILDA, (Briggs et al., 2018) and ≥ 16 for NICOLA. For clarity, we use the term “depression” to indicate clinically significant depressive symptoms throughout this paper. The rationale for using a binary outcome is to focus on whether participants exhibited clinically significant depressive symptoms rather than measuring varying degrees of severity.

### 2.3. Assessment of long-term exposure to outdoor ALAN

The Gateway to Global Aging Data project (g2aging.org) (Lee et al., 2021) linked harmonized ALAN data to participants’ addresses in all surveys. These data were obtained from the Joint Polar-orbiting Satellite System (JPSS) and the Visible and Infrared Imaging Suite (VIIRS) Day Night Band (DNB) technology and processed by the Earth Observation Group to remove pixels affected by sunlight, moonlight, or cloud cover. (Elvidge et al., 2021) We used annual median-masked rasters from version VNL2.1, which reduce background noise (e.g. aurora and biomass burning) and provide ground-level radiance measurements in nanowatts per square centimetre per steradian (nW/cm^2^/sr). These data, available at a 500 m resolution, cover the period from 2012 to 2021.

We linked these data to participants based on their geocoded residential addresses at each survey wave. We then calculated a time-weighted annual mean based on exposure levels in the year preceding the interview and the year of the interview.

### 2.4. Assessment of covariates

Individual-level covariates included age, calendar year at interview, birth cohort (for HRS), race/ethnicity (HRS and ELSA only), sex (male/female), educational attainment, homeownership, wealth, partnership status, working/retirement status, smoking, alcohol, and physical activity levels. We also obtained neighborhood-level characteristics for each participant, including urbanicity (urban or rural) and neighborhood-level socioeconomic status. The covariates were defined and coded in the models as described in Supplementary Table S2.

Additionally, annual mean exposure levels of air pollutants were estimated at the participant’s residence for inclusion in the sensitivity analyses. These included particulate matter with an aerodynamic diameter ≤ 2.5 μm (PM_2.5_), (Shaddick et al., 2018; van Donkelaar et al., 2021) nitrogen dioxide (NO_2_), (Larkin et al., 2023) and Ozone (O_3_) (Becker et al., 2023; Malashock et al., 2022). Residential greenness was also summarized as annual maximum and mean of Normalized Difference Vegetation Index (NDVI), averaged within a 1 km buffer. These environmental factors have been found to be associated with depression. (Zundel et al., 2022; Wang et al., 2024; Fossa et al., 2024; Yoo et al., 2024) Detailed information on air pollution and greenspace assessment can be found in Supplementary Text S2.

### 2.5. Statistical analysis

We used a Poisson regression model with correlated standard errors (Zou and Donner, 2013; Zeger and Liang, 1986; Ballinger, 2004) to examine the association between long-term outdoor ALAN exposure and the presence of depression within each survey separately. Age, calendar time at interview, wealth, ALAN and air pollutant levels were treated as time-varying (except for NICOLA, where only one wave was included), whereas other covariates were based on baseline values. Given that the distribution of ALAN values was highly right-skewed, ALAN exposure was modeled as a categorical variable based on quartile values. Due to the nature of sensitive data being used, it was not possible to pool data from each survey. Therefore, quartile values were derived from population-weighted samples of 10,000 points across the contiguous US, the UK (including Northern Ireland), and Ireland, rather than from the specific cohort locations. The cutoffs for ALAN exposure were 25th percentile: 2.8 nW/cm^2^/s; 50th percentile:11.7 nW/cm^2^/s; 75th percentile: 23.9 nW/cm^2^/s. However, the distribution of these sample points closely tracked the original ALAN distribution in each survey (data not shown). The prevalence ratio (PR) and its 95 % confidence interval (CI) were calculated for each ALAN quartile, using individuals in the lowest quartile as the reference group. Analyses accounted for repeated measures and the complex survey design of each study, including clustering (survey-specific cluster IDs: e.g., ELSA, TILDA: study participant ID; HRS: survey clustering).

We performed models with different levels of adjustment to examine the impact of each covariate on the association between ALAN and having depressive symptoms. In our crude model (Model 1), we adjusted for age, sex, calendar time, race/ethnicity (HRS and ELSA only), and birth cohort (only HRS to reflect the recruitment patterns in this study). In Model 2a, we further adjusted Model 1 for individual socioeconomic status (SES), including educational attainment, wealth, and home ownership at baseline. In Model 2b, we additionally adjusted Model 1 for the area-level multiple deprivation index (MDI). In Model 2c, we further adjusted Model 1 for urbanicity. In Model 3, our main model, we included all covariates mentioned above. For HRS, spatial spline term was included in Model 3. Supplementary Table S2 summarized how the covariates were defined and coded in the models in each survey.

As secondary analyses, we stratified Model 3 by urbanicity and sex to examine whether the association is different by living in urban/rural areas and being men/women, respectively.

We conducted several sensitivity analyses to assess the robustness of our results. First, we investigated the impact of additional adjustments to Model 3, by incorporating individual lifestyle factors such as smoking status, alcohol consumption, and physical activity levels, as well as additional individual sociodemographic factors (partnership and employment status). Additionally, we evaluated the impact of further adjusting Model 3 for season of interview (December-February, March-May, June-August, September-November), long-term exposure to air pollutants and greenspace.

Analyses were conducted separately for each survey using SAS for HRS, R for ELSA, Stata for TILDA, and Python in NICOLA. We considered a two-tailed p-value of less than 0.05 to be statistically significant.

## 3. Results

### 3.1. Sample characteristics

The analytical sample in this study included the total of 39,488 individuals, from 20,868 participants with 48,742 interviews from HRS, 9848 participants with 28,942 interviews from ELSA, 6407 participants with 21,101 interviews from Ireland, and 2725 participants from 2725 interviews from NICOLA. Individuals with missing data in any of the variables were excluded from the analyses:19 % (n = 4,924) in HRS; ~7% (n = 791) in ELSA; ~15 % (n = 1,174) in Ireland; ~27 % (n = 929) in NICOLA. Details of exclusion by covariates can be found in Supplementary Figs. S1-S4.

The prevalence of depression among participants varied across surveys. While approximately 24 % of participants in HRS and ELSA reported experiencing depression during the interviews, the percentage was lower in NICOLA (14 %) and TILDA (8 %). Table 1 summarizes the baseline characteristics of survey participants, according to the presence of depressive symptoms during the interviews. In all surveys, participants with depressive symptoms were more likely to be women, belong to an ethnic minority group (except TILDA and NICOLA not having an ethnic identifier), have education level below college, not to own their home, have lower wealth, and live in deprived areas, compared to those without depressive symptoms.

Table 2 presents the descriptive statistics for the annual mean exposure level to ALAN and its correlation with other air pollutants and greenspace, across the four surveys. HRS had the highest annual mean ALAN exposure level (Mean ± SD: 18.9 ± 9.0 nW/cm^2^/s), while the other three surveys had lower mean level (13.4 ± 12.7 nW/cm^2^/s in ELSA; 10.4 ± 13.4 nW/cm^2^/s in TILDA; 11.6 ± 10.3 nW/cm^2^/s in NICOLA). HRS also had the highest maximum value of 226.1 nW/cm^2^/s while NICOLA had the lowest maximum level of 75.9 nW/cm^2^/s. Across all surveys, NO_2_ showed strong positive correlation with ALAN exposures, particularly in NICOLA [spearman correlation (ρ) = 0.81], PM_2.5_ presented more modest correlation with ALAN (ρ = 0.15 ~ 0.21), while NDVI presented negative correlation (ρ = − 0.52 ~ − 0.86). For O_3_, the correlations with ALAN were negative in HRS and ELSA (ρ = − 0.26 ~ −0.54), but positive and weak in NICOLA (ρ = 0.13).

### 3.2. Association between long-term outdoor ALAN exposure and depression

**Fig. 1.**
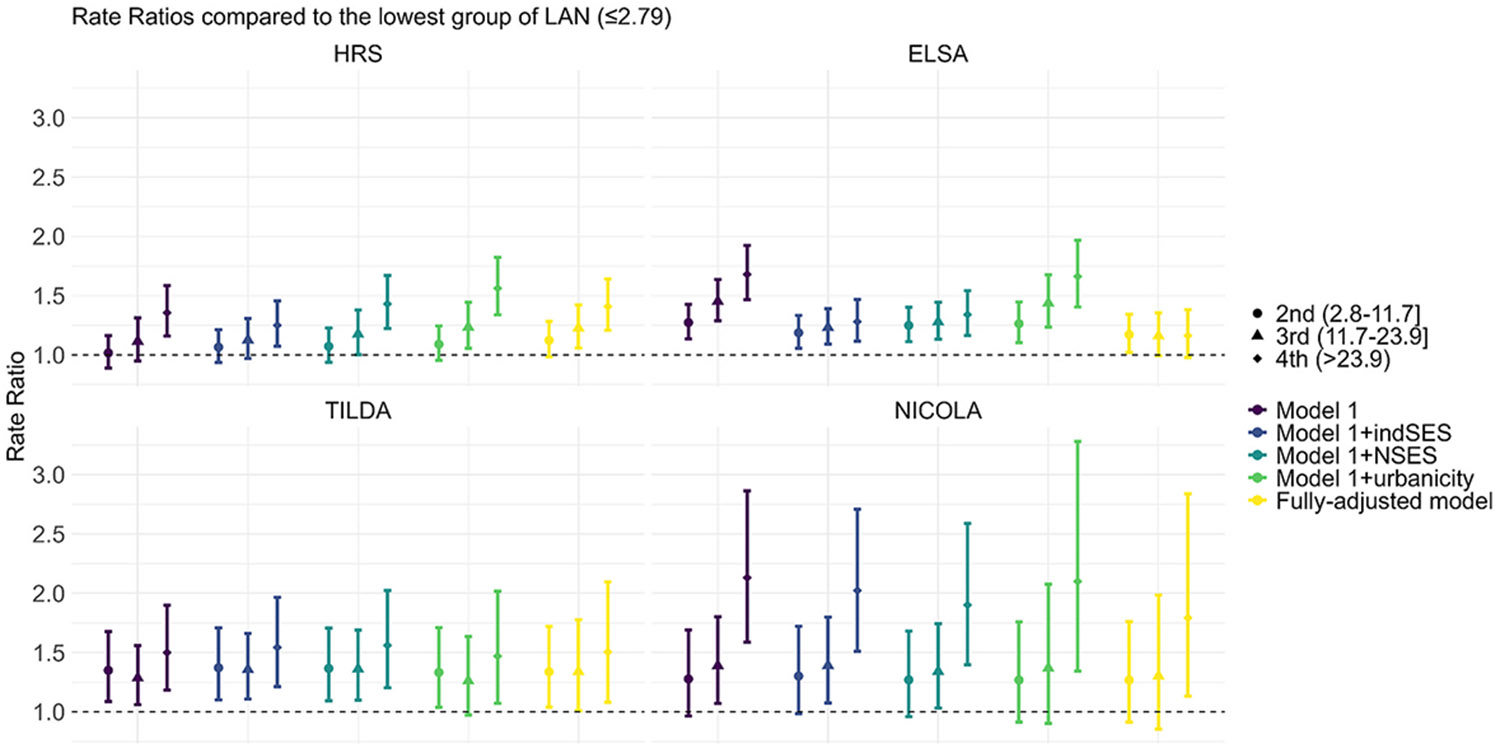
Associations between long-term exposure to outdoor light at night and having depressive symptoms Model1: Age, sex, calendar time, birth cohort (in HRS), race/ethnicity (except TILDA and NICOLA); and Model 1 were adjusted for the following covariates: Individual socioeconomic status (indSES) – educational attainment at baseline, wealth, home ownership at baseline (except NICOLA); neighborhood-level socioeconomic status (NSES); urbanicity. Fully-adjusted models includes all covariates. For HRS, spatial spline term was included in the fully-adjusted model.

Fig. 1 shows the associations between long-term exposure to ALAN and the presence of depressive symptoms across the four surveys, with the numerical results presented in the Supplementary material Table S3. In the fully-adjusted models (Model 3), we generally observed that higher levels of ALAN exposure were associated with the increased prevalence of depression. The PR (95 % CI) for the highest ALAN exposure group (*>*23.9 nW/cm^2^/s) as compared to the reference group (ALAN ≤ 2.8 nW/cm^2^/s) ranged from 1.16 (0.98–1.38) and 1.40 (1.20–1.63) in ELSA and HRS, respectively, to 1.51 (1.08–2.10) and 1.79 (1.13, 2.84) in TILDA and NICOLA, respectively.

The associations in HRS, TILDA, and NICOLA were robust across different adjustments, whereas ELSA showed a noticeable attenuation in effect size, particularly after adjusting for individual- and neighborhood-level SES.

### 3.3. Stratification by urbanicity and sex

Table 3 presents the associations between long-term exposure to outdoor ALAN and the presence of depression, stratified by urbanicity (rural vs. urban areas) across the surveys. The associations by urbanicity varied across countries. In TILDA and ELSA, stronger associations were observed in rural populations compared to urban populations. In the highest ALAN exposure group, rural participants had PRs of 3.07 (95 % CI, 1.43–6.61) in TILDA and 2.55 (95 % CI, 1.53–4.24) in ELSA, compared to those in the lowest exposure group. In HRS, both urban and rural populations showed moderate but significant associations, with a slightly stronger effect observed in urban areas with an PR of 1.29 (95 % CI, 1.04–1.60) in rural populations and 1.47 (95 % CI, 1.13, 1.91) in urban populations in the highest ALAN group. In NICOLA, the estimates were imprecisely estimated, with extremely wide confidence intervals, and cannot be distinguished from the null. It is worth noting that the median ALAN exposure was 1.1 nW/cm^2^/s (25th & 75th percentile: 0–4) for participants living in rural areas and 16.7 nW/cm^2^/s (25th & 75th percentile: 12.3–22.2) in urban areas in Northern Ireland. Stratification combined with grouping based on harmonized ALAN thresholds yielded imbalanced subgroups.

Sex-stratified analyses across ELSA, TILDA, and NICOLA revealed consistent directional associations between LAN exposure and the outcome for both men and women. In all cohorts, higher LAN exposure was associated with increased risk compared to the lowest exposure category. However, statistically significant associations were observed only among men in ELSA, where a clear dose–response relationship was evident across increasing quartiles of exposure. Among women, the associations were generally weaker and less consistent, with significant effects observed only in TILDA. These differences in statistical significance may reflect variations in sample size and statistical power across cohorts and sex groups, rather than true differences in the underlying associations. Overall, the findings suggest that the direction of the association is consistent across sexes and studies, but the strength and precision of the estimates vary (Table 4).

### 3.4. Sensitivity analyses

Further adjustment for air pollutants and greenspace indicated that the associations remained robust (Table S5). However, attenuation of the association was apparent after adjusting for PM_2.5_, NO_2_, and NDVI in TILDA, and NO_2_ and NDVI in NICOLA. However, the observed correlations between ALAN and NO_2_ and NDVI may limit the interpretation of the results. Adjusting for lifestyle factors and additional SES variables led to varying degrees of reduction in effect sizes across cohorts (Supplementary Table S6), but the direction of the observed associations in all cohorts remained consistent.

## 4. Discussion

In these national representative studies of older adults from the US, England, Ireland, and Northern Ireland, we observed that higher long-term exposure to outdoor ALAN was associated with increased prevalence of depression with different strength and precision of the associations. In the highest ALAN exposure group (*>*23.9 nW/cm^2^/s), significant associations were observed in the US, Ireland and Northern Ireland, a moderate but non-significant association in England. Furthermore, urbanicity appeared to modify the associations, with stronger effects observed in rural populations in Ireland and England. In contrast, urban populations in the US showed slightly stronger associations than their rural counterparts.

Although beyond the scope of this paper, it has been suggested that light at night exposure might impact depression by disrupting the circadian rhythm, which in turn may change melatonin secretion and disrupt sleep leading to higher depression. (Ikeno and Yan, 2016; Fonken et al., 2009) Chronic disruption may also affect the hypothalamic–pituitary–adrenal axis, resulting in elevated stress responses, neuroinflammation, and dysregulated cortisol levels, all of which have been linked with mood disorders. (Sălcudean et al., 2025; Samanta and Bagchi, 2024) Additionally, light at nightexposure may reduce sleep quality, affecting emotional regulation and potentially contributing to depressive symptoms. (Ikeno and Yan, 2016).

Limited studies have examined the link between ALAN exposure and depression. Consistent with our analysis, previous studies using satellite data for outdoor ALAN have reported increased depression risk with higher ALAN exposure. This includes evidence from a cross-sectional study in South Korea with 113,119 adults aged 20–59 years with odds ratio (OR) of 1.29 (95 % CI, 1.15–1.46) for depression, defined based on the Korean version of CES-D, in the highest quartile (≥60.4 nW/cm^2^/s) compared to the lowest (≤13.2 nW/cm^2^/s); (Min et al., 2018) a cross-sectional nationally representative study of 10,482 Dutch adults aged 18–65 years reporting 0.5-point (95 % CI, 0.1–0.9) increase in depressive symptom score (range: 0—27), in the highest quintile (21.1–97.4 nW/cm^2^/s) compared to the lowest (<4.6 nW/cm^2^/s); (Helbich et al., 2020) a cohort study using UK biobank data with 298,283 participants presenting hazard ratio of 1.14 (95 % CI, 1.05–1.23) for physician-diagnosed depression in the highest quantile (mean: 28.1 nW/cm^2^/s) compared to the lowest (4.6 nW/cm^2^/s); (Jin et al., 2023) a cross-sectional study of Chinese 6; 445 male veterans aged ≥ 60 years with an OR of 1.49 (95 %CI, 1.15–1.92) for depression, defined based on Chinese version CED-D, in the highest quantile (median: 107.7 nW/cm^2^/s) compared to the lowest (median: 30.7 nW/cm^2^/s); (Zhu et al., 2023) and a Chinese study using a province-level difference-in-differences design with 21,036 adults presenting ORs of 1.05–1.16 across four CES-D items per quartile increment in ALAN. (Yu et al., 2022) However, a direct comparison of the magnitude of the association across these studies and ours is challenging due to differences in outcomes, representativeness of the populations, and levels of ALAN.

Most of these studies adjusted for air pollutants, particularly PM_2.5_ or PM_10_, but only the Dutch study conducted sensitivity analyses adjusting for NO_2_. Given its strong correlation with ALAN (r = 0.85), the Dutch study reported that the ALAN-depression association was not robust to adjustment for NO_2_. Our study similarly found that adjusting for NO_2_ reduced effect sizes in TILDA and NICOLA, but increased estimates in ELSA. This suggests that the association of ALAN with depression may be partially confounded by NO_2_ or other features of the urban setting that co-occur with light sources, such as traffic.

Notably, there are a few studies in smaller samples to investigate indoor ALAN exposure and depressive mood, which may be a better reflection of personal exposures to light and may be less confounded by place. These cross-sectional studies, however, have reported mixed findings. For example, one small study in the U.S. (154 women aged 51–81 years) found no association with dim light exposure before bedtime (median 24 lx, 4 h); (Wallace-Guy et al., 2002) while a Japanese study (516 adults aged ≥ 60 years) identified a significant association with higher-intensity nighttime light exposure (≥5 lx) and prolonged duration of nighttime light exposure (≥10 lx for ≥ 30 min) (Obayashi et al., 2013). A more recent cohort study from Japan confirmed these findings (Obayashi et al., 2018). This highlights the need for further confirmatory research to investigate the isolated effects of light pollution on mental health.

Another important consideration is that ALAN can originate from different sources and carry different implications in urban versus rural settings. To this extent, our study contributes to the literature by being the first to examine urban–rural differences in the association between ALAN exposure and depressive symptoms. While we found evidence that urbanicity was important for these relationships, it is worth noting that we generally found ALAN to be associated with depression in both settings. In Ireland and England, these associations were stronger for rural populations, whereas the opposite was true in the US and Northern Ireland. This could be due to differences in the sources of light in urban and rural settings and correlated exposures or differences in adaptation behaviors to mitigate ALAN exposure, such as using blackout curtains or sleep masks.

This analysis has several strengths. First, we leveraged harmonized exposure data across multiple countries to estimate the associations between ALAN and depression. Our finding of associations between greater ALAN exposures and depression across all settings supports the hypothesis that ALAN is important for mental health. Our inclusion of nationally representative samples from the US, England, Ireland, and Northern Ireland enhances the generalizability of our findings. Finally, we extensively adjusted for both individual- and neighborhood-level characteristics, including air pollutants and greenspace, which are known to be associated with depression. (Zundel et al., 2022; Wang et al., 2024; Fossa et al., 2024; Yoo et al., 2024).

Despite the many strengths of this work, some limitations should be noted. Our ALAN exposure assessment relied on satellite data, which captures outdoor lighting, and failed to account for personal nighttime light exposure from indoor sources such as bedroom lighting, TV screens, or smartphones. Additionally, residual confounding may remain because some pollutant estimates have coarser resolution (e.g., PM_2.5_ at ~1 km and O_3_ at ~11 km). Furthermore, although our specification of NDVI as a maximum greenness over the year within a 1-km buffer are expected to be correlated with other measures, it is possible that we did not select the most ideal measure for this covariate (Browning and Lee, 2017; Klompmaker et al., 2018) and there is some residual confounding by these exposures. We also lacked consistent shift-work measures across surveys, and therefore could not adjust for shift-work directly. Although those who work nights may experience light at night differently than those who work daytime hours, this issue may be more limited in this ≥ 50-year old population, where many participants are retired. In addition, while we adjusted for urbanicity and air pollution, other contextual factors, such as local lighting ordinances, practices on shielding of nighttime lighting, and other socioeconomic variables—may confound the observed associations. It is also important to note that we restricted our study to high-income countries of the Gateway to Global Aging Data project, potentially limiting generalizability of our findings to low- and middle-income countries, where patterns of urban structures and light pollution levels may differ. Future research should aim to explore more individual-level light exposure assessment and these associations in diverse global settings to increase the understanding of the impact of ALAN on mental health.

## 5. Conclusion

In this longitudinal study, which drew on nationally representative surveys from four countries, we found that long-term exposure to outdoor ALAN was associated with higher risk of depressive symptoms in older adults. Stratified analyses indicated that these associations varied by urbanicity and countries. We also found that other environmental characteristics, such as air pollution, may also act as an important confounder, complicating efforts to isolate the independent effects of ALAN on depression. To overcome these challenges, future research should incorporate individual-level light exposure assessments – such as wearable sensors or residential light measurements – or employ advanced modelling approach to better understand the mechanisms linking ALAN to mental health.

## Supplementary Material

Supplementary material

## Figures and Tables

**Table 1:** Baseline Characteristics of included HRS, ELSA, TILDA, and NICOLA participants by having depression.

Survey (total individual, n)	HRS (20,868)		ELSA (9,848)		TILDA (6,407)		NICOLA (2,725)	
Ever depressed^a^, n (%)	Yes:5,069 (24.3)	No: 15,799 (75.7)	Yes: 2,385 (24.2)	No: 7,463 (75.8)	Yes: 514 (8.0)	No:5,865 (92.0)	Yes: 376 (14.0)	No: 2349 (86)
Age (year), mean ± SD	64.2 ± 4.5	64.3 ± 4.6	67.0 ± 10.6	65.8 ± 10.0	63.3 ± 9.3	64.4 ± 9.6	62.5 ± 9.2	64.1 ± 9.0
Female, (%)	61.7	51.5	66.9	52.9	64.2	55.1	61.7	50.8
Ethnicity^b^, (%)								
Non-Hispanic white^b^	67.2	77.9	93.7	96.3	–	–	–	–
Black	14.0	10.0	–	–	–	–	–	–
Hispanic white	7.5	4.7	–	–	–	–	–	–
Others^b^	11.3	7.4	5.2	3.7	–	–	–	–
Having higher education^c^, (%)	44.6	58.0	23.0	33.4	27.6	34.8	18.9	25.3
Own home, (%)	56.6	72.0	70.6	86.9	63.0	66.0	–	–
Wealth, (%)								
1st (Lowest quintile for HRS)	19.1	17.9	53.5	32.4	45.5	33.9	52.7	45.6
2nd	–	–	29.0	34.9	30.4	33.4	25.3	19.5
3rd (Highest quintile for HRS)	9.7	21.8	17.6	32.6	24.1	32.7	22.1	35.0
Area-level deprivation, (%)								
1st	–	–	25.1	35.2	25.1	36.8	29.3	36.3
2nd	–	–	31.0	33.9	38.1	32.6)	29.5	33.5
3rd	–	–	43.9	30.9	36.8	30.6	41.2	30.2
Area-level deprivation, mean ± SD	−0.01 (0.41)	−0.21 (0.44)	–	–	–	–	–	–
Living in Urban, (%)	48.8	50.6	78.2	74.1	59.3	51.6	66.5	58.0

a Indicated whether the participants was classified as having depressive symptoms based on a survey-specific cutoff of the CED-S score in any of the included waves of the study.

b Difference in ethnicity was considered only in HRS and ELSA: Non-histpanic white, black, hispanic white, or other in HRS; white or non-white in ELSA.

c Higher education equal or above college level education.

**Table 2:** Descriptive statistics of long-term exposure to light at night in the included studies of HRS, ELSA, TILDA, and NICOLA.

Study	Mean ± SD	IQR	Min	Percentile			Max	Spearman’s correlation with the below air pollutant or greenspace			
				25th	50th	75th		PM_2.5_	NO_2_	O_3_	NDVI
HRS, nW/cm^2^/s	18.9 ± 9.0	22.8	0.0	4.14	12.6	27.0	226.1	0.37	0.57	−0.44	−0.52
ELSA, nW/cm^2^/s	13.4 ± 12.7	13.8	0.0	4.6	10.5	18.4	169.3	0.15	0.69	−0.26	−0.71
TILDA, nW/cm^2^/s	10.4 ± 13.4	18.7	0.0	0.2	4.5	18.9	148.4	0.22	0.81	−0.40	−0.48
NICOLA, nW/cm^2^/s	11.6 ± 10.3	16.3	0.0	2.1	10.3	18.4	75.9	0.21	0.81	0.13	−0.86

**Table 3:** Prevalence ratios (95% confidence intervals) of depression associated with long-term exposure to outdoor light at night and having depressive symptoms by urbanicity.

LAN	HRS	ELSA	TILDA	NICOLA
**Rural**				
1st (0—2.8)	Reference	Reference	Reference	Reference
2nd (2.8–11.7)	1.12 (0.95, 1.32)	1.19 (1.02, 1.40)	1.33 (1.05, 1.69)	1.29 (0.92, 1.82)
3rd (11.7—23.9)	1.29 (1.06, 1.58)	1.14 (0.74, 1.76)	1.73 (1.06, 2.82)	0.84 (0.21, 3.38)
4th (*>*23.9)	1.29 (1.04, 1.60)	2.55 (1.53, 4.24)	3.07 (1.43, 6.61)	no observation
**Urban**				
1st (0—2.8)	Reference	Reference	Reference	Reference
2nd (2.8–11.7)	1.13 (0.89, 1.42)	1.08 (0.82, 1.42)	1.30 (0.95, 1.77)	1.76 (0.25, 12.34)
3rd (11.7—23.9)	1.16 (0.90, 1.49)	1.08 (0.82, 1.42)	1.27 (0.94, 1.71)	1.82 (0.26, 12.68)
4th (*>*23.9)	1.47 (1.13, 1.91)	1.07 (0.80, 1.42)	1.43 (1.05, 1.94)	2.52 (0.36, 17.69)

Associations were from fully-adjusted models adjusted for age, sex, calendar time, birth cohort (in HRS), race/ethnicity (except TILDA and NICOLA), individual socioeconomic status, including educational attainment at baseline, wealth, home ownership at baseline (except NICOLA), and neighborhood-level socioeconomic status. For HRS, spatial spline term was included in the fully-adjusted model.

## Data Availability

The authors do not have permission to share data.

## Appendix A. Supplementary data

Supplementary data to this article can be found online at https://doi.org/10.1016/j.envint.2025.109886.
