# Supplementary material for: Artificial outdoor light at night and depression in older adults in the USA, England, Northern Ireland, and Ireland
Source: Environ Int. Author manuscript; Available in PMC 2026 Mar 13. (PMC12987635; doi:10.1016/j.envint.2025.109886)
Supplement: Supplementary material [file NIHMS2145061-supplement-Supplementary_material.docx]

**Supplementary material**

**Artificial outdoor light at night and depression in older adults in the USA, England, Northern Ireland, and Ireland**

Rina So, Jennifer D’Souza, Joanne Feeney, Hüseyin Küçükali, Kayleigh P. Keller, Giorgio Di Gessa, Joanna Valson, Ruth F. Hunter, Anne Nolan, Sinead Mc Loughlin, Jinkook Lee, Sara D. Adar, Paola Zaninotto

**Text S1. Description of studies**

The Health and Retirement Study (HRS) is a nationally representative biennial longitudinal survey of people aged 51 years and older that began in 1992 and currently includes more than 37,000 individuals.^1^ Since 1992 HRS has grown to represent all Americans aged 50 years and older. The sample selection was based on a multistage area probability design involving geographical stratification and clustering. Baseline data collection is through face-to-face interviews and follow-up interviews rotate between face-to-face and telephone administration (for those aged less than 80 years). National representation of this population is maintained over time using a steady state design which adds a new cohort of persons entering their 50’s every 6 years. HRS protocols are reviewed and approved by the Health Sciences and Behavioral Sciences Institutional Review Board at the University of Michigan, and all participants provide informed consent prior to participation, in line with ethical research standards.

The English Longitudinal Study of Ageing (ELSA), is also an open-access, nationally representative, biennial longitudinal survey of those aged 50 years and older living in private households in England that began in 2002/2003.^2^ The sample was drawn from participants in the Health Survey for England (HSE), an annual cross-sectional survey that is designed to monitor the health of the general population.^2^ For the first wave (2002/2003), participants were recruited from the HSE by using a two-stage stratified random sampling process. Data were also collected through face-to-face interviews at each wave. Comparisons of the sociodemographic characteristics of participants against results from the 2011 national census indicate that the sample was broadly representative of the English population.^2^ Ethical approval was obtained from the NRES Committee South Central – Berkshire, and informed consent was obtained from all individual participants included in the study.

The Irish Longitudinal Study on Ageing (TILDA) is a large, nationally representative, prospective cohort study of community-dwelling adults aged 50 years and older in Ireland, along with their spouses of any age.^3^ The study was established to investigate the health, social, and economic circumstances of older people, with baseline interviews (Wave 1) conducted between October 2009 and February 2011, enrolling 8507 participants. The sampling frame was the Irish Geodirectory, a comprehensive register of all residential addresses in the Republic of Ireland, from which participants were randomly selected using the RANSAM multi-stage probability procedure to ensure equal probability of household selection. Data collection at each wave comprises three components: a computer-assisted personal interview (CAPI) in participants’ homes, a self-completion questionnaire, and a detailed health assessment at a dedicated health centre or, for less mobile participants, in their homes. Successive biennial waves have since been conducted, covering a wide range of health, social, and economic domains. Ethical approval was obtained from the Trinity College Dublin Faculty of Health Sciences Research Ethics Committee, and written informed consent was provided by all participants, in line with the Declaration of Helsinki.

The Northern Ireland Cohort for the Longitudinal Study of Ageing (NICOLA) (26) is Northern Ireland’s largest health and social care cohort, established to investigate the biological, social, and economic determinants of ageing. ^4^ The baseline survey (Wave 1) recruited 8478 community-dwelling adults aged 50 years and older, along with their spouses or partners of any age, between December 2013 and March 2016. Participants were selected using a randomized, stratified sample of Northern Ireland addresses drawn from the General Practitioner Register Database, supplemented with Land and Property Services address data, and were invited to complete a computer-assisted personal interview (CAPI), a self-completion questionnaire, and a detailed health assessment carried out in a clinical facility or at home. A follow-up (Wave 2) took place between 2017 and 2019 with a 73% response rate (n = 6152). Biennial interviews and quadrennial health assessments are planned for future waves. The study is harmonized with ELSA and TILDA, facilitating cross-country comparisons, and also includes measures specific to Northern Ireland, such as the impact of the Troubles. NICOLA received ethical approval from the School of Medicine, Dentistry and Biomedical Sciences, Queen’s University Belfast, and written informed consent was obtained from all participants

**Table S1. Summary of the Center for Epidemiologic Studies Depression Scale (CES-D) items used in the original and short version.**

| **Item number** | 20-items | 8-items | 8-items |
| --- | --- | --- | --- |
| **Score range** | 0-60 | 0-24 | 0-8 |
| **Cut-off reflecting clinical depression** | ≥16 | ≥9 | ≥4 |
| **Item/Response** | Frequency^a^ | Frequency^a^ | Binary |
| 1. I was bothered by things that usually don’t bother me. | 0-3 |  |  |
| 2. I did not feel like eating; my appetite was poor. | 0-3 |  |  |
| 3. I felt that I could not shake off the blues, even with the help from family or friends. | 0-3 |  |  |
| 4. I felt that I was just as good as other people. | 0-3 ^b^ |  |  |
| 5. I had trouble keeping my mind on what I was doing. | 0-3 |  |  |
| 6. I felt depressed. | 0-3 | 0-3 | No=0; Yes=1 |
| 7. I felt that everything I did was an effort. | 0-3 | 0-3 | No=0; Yes=1 |
| 8. I felt hopeful about the future. | 0-3 ^b^ |  |  |
| 9. I thought my life had been a failure. | 0-3 |  |  |
| 10. I felt fearful. | 0-3 |  |  |
| 11. My sleep was restless. | 0-3 | 0-3 | No=0; Yes=1 |
| 12. I was happy. | 0-3 ^b^ | 0-3 ^b^ | No=0; Yes=1 ^b^ |
| 13. I talked less than usual. | 0-3 |  |  |
| 14. I felt lonely. | 0-3 | 0-3 | No=0; Yes=1 |
| 15. People were unfriendly. | 0-3 |  |  |
| 16. I enjoyed life. | 0-3 ^b^ | 0-3 ^b^ | No=0; Yes=1 ^b^ |
| 17. I had crying spells. | 0-3 |  | No=0; Yes=1 |
| 18. I felt sad. | 0-3 | 0-3 | No=0; Yes=1 |
| 19. I felt that people disliked me. | 0-3 |  |  |
| 20. I could not get “going”. | 0-3 | 0-3 | No=0; Yes=1 |

^a^Frequency responses were: 0= Rarely or none of the time (< 1 day/week); 1 = Some or a little of the time (1-2 days/week); 2 = Occasionally or a moderate amount of time (3-4 days/week); 3 = Most or all of the time (5-7 days/week). ^b^ Positive items with reversed scoring.

**Table S2. Summary of covariates included in the analyses by surveys**

| **Variables** | **HRS** | **ELSA** | **TILDA** | **NICOLA** |
| --- | --- | --- | --- | --- |
| **Age****, continuous** | Age in year at interview | Age in year at interview | Age in year at interview | Age in year at interview |
| **Calendar time, continuous** | Date at interview | Year and month at interview | Year of interview | Interview year |
| **Race/ethnicity, categorical** | Non-Hispanic White, Black, Hispanic, Other/Missing | White, non-white | n/a | n/a |
| **Education attainment at baseline, categorical** | < High school, High school, Some college, College+ | Higher education (>A-level), not, foreign or else | Higher education (≥tertiary) or not | Higher education or not(> 5 from the below list. 1. Some primary (not complete), 2. Primary or equivalent, 3. GCSE/Intermediate/junior/group certificate or equivalent, 4. A-level/Leaving certificate or equivalent, 5. Diploma/certificate, 6. Primary degree, 7. Postgraduate/higher degree  ) |
| **Homeownership at baseline, categorical** | Yes or no | Yes or no | Yes or no | n/a |
| **Wealth, categorical** | Wealth at baseline, including value of primary residence (quintiles) | Wealth at each wave (time-varying) without pension and housing value (tertile) | Non-housing wealth at wave 1 | Wealth at baseline (savings + investments - debts) |
| **Having partner, categorical** | Having partner or not | Having a partner or not; having a partner-Married/Living with a partner; not having a partner-single/separated/divorced/widowed | Having a partner or not; having a partner- Married/Living with a partner as if married; not having a partner- Single (never married)/Separated/ Divorced/Widowed | Having a partner or not; having a partner- Married/Living with a partner as if married; not having a partner- Single (never married)/Separated/ Divorced/Widowed |
| **Working or retirement status, categorical** | Working for pay at baseline (yes or no); retired at baseline (yes or no) | Working status (yes or no) | Employed/not employed/retired | Employed or not (employed – Employ/Self-employed; Not employed-retired/ unemployed/ permanently sick or disabled/ looking after home or family/ in education or training) |
| **Smoking status, categorical** | Ever smoked (yes or no) | Never smoked, previous smoker, current smoker | Never smoked, previous smoker, current smoker | Ever smoked or not |
| **Alcohol drinking status, categorical** | Heavy drinking at baseline (yes or no) | Drink 5-7 days a week, or less than 5 days a week | Problem drinking (defined by the CAGE scale) | Ever used alcohol or not |
| **Physical activity levels, categorical** | N/A | no activity on a weekly basis, only mild activity at least once a week, or at least moderate to vigorous activity at least once a week | Physical activity level (low, moderate, high) based on the International Physical Activity Questionnaire (IPAQ) | Any moderate to vigorous physical activity or not |
| **Urbanicity, categorical** | Urban or suburban +rural^a^ | Urban or rural^b^ | Urban or rural^c^ | Urban or rural^b^ |
| **Neighborhood-level socioeconomic status, , categorical except for HRS** | Continous; using a principal components analysis of tract-level data from the American Community Survey reflecting the proportion of those with a bachelor’s degree, proportion of those with managerial occupation, median home value, median household income, proportion of those with a high school degree or above, proportion of those with interest/dividend/rental income, and proportion of those with a household income >$50,000. Higher (or positive values) indicate greater disadvantage. | Multiple Deprivation Index (tertiles) | Multiple Deprivation Index (tertiles) | Northern Ireland Multiple Deprivation Measure (tertile) |

^a^ [https://hrs.isr.umich.edu/data-products/restricted-data/available-products/9706](https://eur02.safelinks.protection.outlook.com/?url=https%3A%2F%2Fhrs.isr.umich.edu%2Fdata-products%2Frestricted-data%2Favailable-products%2F9706&data=05%7C02%7Crina.so%40sund.ku.dk%7C85a07f4e0a61415598f908dd0fc0c7ba%7Ca3927f91cda14696af898c9f1ceffa91%7C0%7C0%7C638684042864180353%7CUnknown%7CTWFpbGZsb3d8eyJFbXB0eU1hcGkiOnRydWUsIlYiOiIwLjAuMDAwMCIsIlAiOiJXaW4zMiIsIkFOIjoiTWFpbCIsIldUIjoyfQ%3D%3D%7C0%7C%7C%7C&sdata=3MiLY2emv9nTyoA01Md6RAZFRA6uYDl6lRozC96JHGk%3D&reserved=0)

^b^ <https://www.gov.uk/government/collections/rural-urban-classification>

^c^ The interviewer coded urbanicity as Dublin, other large town/city (=urban), or rural, based on the respondent’s residence.

**Text S2. Air pollution and greenspace assessment.**

Particulate matter with an aerodynamic diameter ≤2.5µm (PM_2.5_) estimates were obtained from the V5.GL.04 version of a global spatiotemporal model developed by van Donkelaar and colleagues.^5,6^ This model integrates data from multiple sources, including ground-level measurement stations, satellites observations, meteorological and chemical transport models, and area-level characteristics, to estimate outdoor concentrations of PM_2.5_ at approximately 1km×1km resolution. The geographically weighted regression approach used in this model has strong agreement with ground level monitors (cross-validated R^2^=0.90) and allows for estimation of PM_2.5_ levels at participant addresses, even in areas with limited or absent ground monitoring.

Annual average outdoor nitrogen dioxide (NO_2_) concentrations were estimated for each participant based on their residential histories using model predictions available at a resolution of approximately 500m×500m for the period 2005 to 2019.^7^ The estimates were predicted by daily land use regression models derived from 8,250 monitors primarily in Europe, North America, and Asia, as well as more limited monitoring coverage in Africa, Oceana, and South America. Cross-validation indicated that the model explained 63% of the variability and had a root mean square error of 4.4 ppb for the annual average estimates.

For ozone, annual averages concentrations (in parts per billion, ppb) were estimated at participant addresses using a global model of ozone^8^ that estimated levels at a 11km×11km resolution for years 1990-2017. This model combined a comprehensive data of ground level ozone monitoring data and multiple global atmospheric models using a Bayesian Maximum Entropy model with a Regionalized Air Quality Model Performance (RAMP) bias correction to address differential model performance around the globe. Originally designed for the Institute for Health Metrics and Evaluation Global Burden of Disease project, these estimates have been further refined for the worldwide estimates of attributable mortality.^9^

Exposure to greenspace were estimated using Normalized Difference Vegetation Index (NDVI) with data sourced from the Google Earth Engine and derived from 250 meter resolution images captured by the Moderate Resolution Imaging Spectroradiometer aboard NASA's Terra satellite (MODIS-Terra, version 6.1 of MOD13Q1 data). NDVI values range from -1 to 1, where higher values indicate dense vegetation, while lower or negative values correspond to surfaces such as water, pavement, and barren land. For our analysis, we calculated the annual maximum and mean of NDVI, averaged within a 1km buffer around participants’ locations. We selected the annual maximum of NDVI for the year to capture the presence of vegetation, irrespective of its greenness at a specific time or the duration of its greenness throughout the year, and averaged it within 1km to approximate neighborhood context, which has been shown to capture a common distance of walking trips.^10^

**Figure S1. Participants exclusion from the study in HRS**

**Exclusion criteria**

25,792 subjects

25,789 subjects

**Survey participants**

3 subjects: missing on covariates

1,134 subjects: missing on CES-D

3,787 subjects: missing on exposure

24,655 subjects

20,868 subjects

**Figure S2. Participants exclusion from the study in ELSA.**

**Exclusion criteria**

10,639 subjects

9,848 subjects

10,208 subjects

**Survey participants**

430 subjects: missing on covariates and exposure*

360 subjects: missing on CES-D

**Figure S3. Participants exclusion from the study in TILDA.**

**Exclusion criteria**

7,581 subjects

6,407 subjects

7,487 subjects

**Survey participants**

94 subjects: missing on CES-D

1080 subjects: missing on covariates and exposure*

**Figure S4. Participants exclusion from the study in NICOLA.**

**Exclusion criteria**

3,654 subjects

2,725 subjects

3,048 subjects

**Survey participants**

606 subjects: missing on CES-D

323 subjects: missing on covariates

O_3_: 242

Education: 65

PM_2.5_: 27

**Table S3. Estimates prevalence ratios of depression related to long-term exposure to outdoor light at night**

| **LAN** | **Model 1** | **Model 1 + indSES** | **Model 1 + NSES** | **Model1 + urbanicity** | **Fully-adjusted model** |
| --- | --- | --- | --- | --- | --- |
| **HRS** |  |  |  |  |  |
| 1^st^ (0- 2.79) | Reference | Reference | Reference | Reference | Reference |
| 2^nd^ (2.79-11.7) | 1.02 (0.89, 1.16) | 1.07 (0.94, 1.21) | 1.07 (0.94, 1.23) | 1.09 (0.96, 1.24) | 1.03 (0.90, 1.17) |
| 3^rd^ (11.7 -23.9) | 1.12 (0.95, 1.31) | 1.13 (0.97, 1.31) | 1.18 (1.00, 1.38) | 1.23 (1.06, 1.44) | 1.14 (0.98, 1.34) |
| 4^th^ (>23.9) | 1.36 (1.16, 1.58) | 1.25 (1.07, 1.45) | 1.43 (1.22, 1.67) | 1.56 (1.34, 1.82) | 1.40 (1.20, 1.63) |
| **ELSA** |  |  |  |  |  |
| 1^st^ (0- 2.79) | Reference | Reference | Reference | Reference | Reference |
| 2^nd^ (2.79-11.7) | 1.27 (1.14, 1.43) | 1.19 (1.06, 1.33) | 1.25 (1.11, 1.40) | 1.26 (1.10, 1.45) | 1.17 (1.02, 1.34) |
| 3^rd^ (11.7 -23.9) | 1.45 (1.29, 1.64) | 1.23 (1.09, 1.39) | 1.28 (1.13, 1.44) | 1.44 (1.23, 1.67) | 1.16 (0.99, 1.36) |
| 4^th^ (>23.9) | 1.68 (1.47, 1.92) | 1.28 (1.12, 1.47) | 1.34 (1.16, 1.54) | 1.66 (1.40, 1.97) | 1.16 (0.98, 1.38) |
| **TILDA** |  |  |  |  |  |
| 1^st^ (0- 2.79) | Reference | Reference | Reference | Reference | Reference |
| 2^nd^ (2.79-11.7) | 1.35 (1.09, 1.68) | 1.37 (1.10, 1.71) | 1.37 (1.09, 1.71) | 1.33 (1.04, 1.71) | 1.34 (1.04, 1.72) |
| 3^rd^ (11.7 -23.9) | 1.29 (1.06, 1.56) | 1.36 (1.11, 1.66) | 1.36 (1.10, 1.69) | 1.26 (0.97, 1.64) | 1.34 (1.01, 1.78) |
| 4^th^ (>23.9) | 1.50 (1.18, 1.90) | 1.54 (1.21, 1.97) | 1.56 (1.20, 2.02) | 1.47 (1.07, 2.02) | 1.51 (1.08, 2.10) |
| **NICOLA** |  |  |  |  |  |
| 1^st^ (0- 2.79) | Reference | Reference | Reference | Reference | Reference |
| 2^nd^ (2.79-11.7) | 1.28 (0.97, 1.69) | 1.3 (0.98, 1.72) | 1.27 (0.96, 1.68) | 1.27 (0.91, 1.76) | 1.27 (0.91, 1.76) |
| 3^rd^ (11.7 -23.9) | 1.39 (1.07, 1.80) | 1.39 (1.08, 1.8) | 1.34 (1.03, 1.74) | 1.37 (0.90, 2.08) | 1.30 (0.86, 1.98) |
| 4^th^ (>23.9) | 2.13 (1.59, 2.86) | 2.02 (1.51, 2.71) | 1.90 (1.40, 2.59) | 2.10 (1.34, 3.28) | 1.79 (1.13, 2.84) |

Model1: Age, sex, calendar time, birth cohort (in HRS), race/ethnicity (except TILDA and NICOLA); and Model 1 were adjusted for the following covariates: Individual socioeconomic status (indSES) - educational attainment at baseline, wealth, home ownership at baseline (except NICOLA); neighborhood-level socioeconomic status (NSES); urbanicity. Fully-adjusted models includes all covariates. For HRS, spatial spline term was included in the fully-adjusted model.

**Table S4. Prevalence ratios (95% confidence intervals) of depression associated with long-term exposure to outdoor light at night and having depressive symptoms by sex**

| **LAN (nW/cm^2^/s)** | **ELSA** | **TILDA** | **NICOLA** |
| --- | --- | --- | --- |
| **Male** |  |  |  |
| 1^st^ (0- 2.8 ) | Reference | Reference | Reference |
| 2^nd^ (2.8-11.7) | 1.24 (1.01, 1.54) | 1.00 (0.71, 1.41) | 1.28 (0.76, 2.15) |
| 3^rd^ (11.7 -23.9) | 1.35 (1.08, 1.69) | 1.34 (0.82, 2.17) | 1.33 (0.67, 2.62) |
| 4^th^ (>23.9) | 1.36 (1.05, 1.75) | 1.48 (0.85, 2.57) | 1.90 (0.90, 4.03) |
| **Female** |  |  |  |
| 1^st^ (0- 2.8) | Reference | Reference | Reference |
| 2^nd^ (2.8-11.7) | 1.15 (1.00, 1.32) | 1.53 (1.08, 2.17) | 1.26 (0.83, 1.91) |
| 3^rd^ (11.7 -23.9) | 1.09 (0.94, 1.26) | 1.34 (0.95, 1.89) | 1.28 (0.75, 2.18) |
| 4^th^ (>23.9) | 1.08 (0.91, 1.28) | 1.51 (1.00, 2.27) | 1.74 (0.97, 3.10) |

Associations were from fully-adjusted models adjusted for age, calendar time, ethnicity (ELSA), individual socioeconomic status, including educational attainment at baseline, wealth, home ownership at baseline (except NICOLA), and neighborhood-level socioeconomic status; urbanicity.

Please note: the results for HRS are not available at the time of revision of this work.

**Table S5. Prevalence ratios (95% confidence intervals) of having depression symptoms associated with light at night, adjusting for air pollution and greenness**

| **LAN, quantile** | **Main model** | **+ PM_2.5_** | **+ NO_2_** | **+ O_3_** | **+ annual max NDVI** | **+ annual mean NDVI** |
| --- | --- | --- | --- | --- | --- | --- |
| **HRS** |  |  |  |  |  |  |
| 1^st^ (0- 2.79) | Reference | Reference | Reference | Reference | Reference | NA^b^ |
| 2^nd^ (2.79-11.7) | 1.12 (0.98, 1.28) | 1.14 (1.00, 1.13) | 1.12 (0.98, 1.29) | 1.12 (0.98, 1.28) | 1.14 (1.01, 1.29) | NA^b^ |
| 3^rd^ (11.7 -23.9) | 1.23 (1.06, 1.42) | 1.26 (1.08, 1.47) | 1.23 (1.05, 1.43) | 1.22 (1.04, 1.42) | 1.24 (1.08, 1.43) | NA^b^ |
| 4^th^ (>23.9) | 1.41 (1.21, 1.64) | 1.47 (1.24, 1.73) | 1.41 (1.20, 1.66) | 1.39 (1.18, 1.65) | 1.38 (1.19, 1.59) | NA^b^ |
| **ELSA** |  |  |  |  |  |  |
| 1^st^ (0- 2.79) | Reference | Reference | Reference | Reference | Reference | Reference |
| 2^nd^ (2.79-11.7) | 1.17 (1.02, 1.34) | 1.17 (1.02, 1.34) | 1.19 (1.03, 1.36)* | 1.17 (1.02, 1.34) | 1.17 (1.02, 1.35)* | 1.17 (1.01, 1.34)*^c^ |
| 3^rd^ (11.7 -23.9) | 1.16 (0.99, 1.36) | 1.16 (0.99, 1.35) | 1.19 (1.02, 1.40)* | 1.15 (0.99, 1.35) | 1.16 (0.99, 1.37)* | 1.14 (0.96, 1.34)* ^c^ |
| 4^th^ (>23.9) | 1.16 (0.98, 1.38) | 1.17 (0.98, 1.39) | 1.22 (1.01, 1.47)* | 1.15 (0.97, 1.37) | 1.17 (0.97, 1.41)* | 1.11 (0.91, 1.35)* ^c^ |
| **TILDA** |  |  |  |  |  |  |
| 1^st^ (0- 2.79) | Reference | Reference | Reference | Reference | Reference | Reference |
| 2^nd^ (2.79-11.7) | 1.41 (1.04, 1.90) | 1.06(0.89, 1.25) | 1.04(0.88, 1.24)^a^ | NA | 1.06(0.89, 1.25) | 1.34 (1.04, 1.74) |
| 3^rd^ (11.7 -23.9) | 1.44 (1.02, 2.04) | 1.29(1.04, 1.60) | 1.24(0.98, 1.56)^a^ | NA | 1.31(1.06, 1.63) | 1.35 (0.98, 1.85) |
| 4^th^ (>23.9) | 1.58 (1.06, 2.36) | 1.34(1.07, 1.69) | 1.25(0.95, 1.65) ^a^ | NA | 1.41(1.12, 1.78) | 1.52 (1.01, 2.28) |
| **NICOLA** |  |  |  |  |  |  |
| 1^st^ (0- 2.79) | Reference | Reference | Reference | Reference | Reference | Reference |
| 2^nd^ (2.79-11.7) | 1.27 (0.91, 1.76) | 1.27 (0.92, 1.77) | 1.14 (0.81, 1.59)* | 1.27 (0.91, 1.76) | 1.23 (0.88, 1.71)* | 1.19 (0.85, 1.66)* |
| 3^rd^ (11.7 -23.9) | 1.30 (0.86, 1.98) | 1.32 (0.87, 2.02) | 1.05 (0.67, 1.65)* | 1.3 (0.85, 1.98) | 1.17 (0.75, 1.83)* | 1.08 (0.69, 1.70)* |
| 4^th^ (>23.9) | 1.79 (1.13, 2.84) | 1.83 (1.15, 2.91) | 1.32 (0.78, 2.21)* | 1.79 (1.13, 2.84) | 1.45 (0.85, 2.48)* | 1.29 (0.75, 2.22)* |

*correlation with LAN is high

^a^ n= 21,093 interviews

^b^Analyses is not available at the time of revision.

^c^ n= 28,229 interviews

Associations were from fully-adjusted models adjusted for age, sex, calendar time, birth cohort (in HRS), race/ethnicity (except NICOLA), individual socioeconomic status, including educational attainment at baseline, wealth, home ownership at baseline (except NICOLA), neighborhood-level socioeconomic status, urbanicity. For HRS, spatial spline term was included in the fully-adjusted model.

Note: TILDA does not have data on ozone.

**Table S6. prevalence ratios (95% confidence intervals) of depression associated with long-term outdoor light at night, adjusting for additional individual socioeconomic status and lifestyle factors**

| Cohort | N, participants | Model | PR (95% CI) 2^nd^ (2.79-11.7) | PR (95% CI) 3^rd^ (11.7 -23.9) | PR (95% CI) 4^th^ (>23.9) |
| --- | --- | --- | --- | --- | --- |
| HRS | 20,868 | Main model | 1.12 (0.98, 1.28) | 1.23 (1.06, 1.42) | 1.41 (1.21, 1.64) |
|  |  | Main Model +lifestyle factor | 1.11 (0.97, 1.27) | 1.21 (1.04, 1.40) | 1.39 (1.19, 1.62) |
|  | 20,868 | Main model | 1.12 (0.98, 1.28) | 1.23 (1.06, 1.42) | 1.41 (1.21, 1.64) |
|  |  | Main Model +additional indSES | 1.07 (0.95, 1.20) | 1.13 (0.99, 1.29) | 1.27 (1.10, 1.46) |
|  | 20,868 | Main model | 1.12 (0.98, 1.28) | 1.23 (1.06, 1.42) | 1.41 (1.21, 1.64) |
|  |  | Main Model +season | 1.12 (0.98, 1.28) | 1.23 (1.06, 1.43) | 1.42 (1.22, 1.65) |
| ELSA | 9,258 | Main model | 1.15 (0.99, 1.33) | 1.10 (0.93, 1.30) | 1.11 (0.92, 1.34) |
|  |  | Main Model +lifestyle factor | 1.13 (0.97, 1.31) | 1.07 (0.91, 1.27) | 1.06 (0.88, 1.28) |
|  | 9,848 | Main model | 1.17 (1.02, 1.34) | 1.16 (0.99, 1.36) | 1.16 (0.98, 1.38) |
|  |  | Main Model +additional indSES | 1.14 (1.00, 1.31) | 1.11 (0.95, 1.30) | 1.09 (0.92, 1.29) |
|  | 9,848 | Main model | 1.17 (1.02, 1.34) | 1.16 (0.99, 1.36) | 1.16 (0.98, 1.38) |
|  |  | Main Model +season | 1.17 (1.02, 1.34) | 1.16 (0.99, 1.36) | 1.16 (0.98, 1.38) |
| TILDA | 5,864 | Main model | 1.39 (1.13, 1.72) | 1.39 (1.10, 1.76) | 1.62 (1.27, 2.07) |
|  |  | Main Model +lifestyle factor | 1.02(0.84, 1.23) | 1.25(0.98, 1.58) | 1.28(0.99, 1.65) |
|  | 6,407 | Main model | 1.41 (1.04, 1.90) | 1.44 (1.02, 2.04) | 1.58 (1.06, 2.36) |
|  |  | Main Model +additional indSES | 1.02(0.86, 1.20) | 1.19(0.96, 1.47) | 1.26(1.01, 1.58) |
|  | 6,407 | Main model | 1.41 (1.04, 1.90) | 1.44 (1.02, 2.04) | 1.58 (1.06, 2.36) |
|  |  | Main Model +season | 1.05(0.89, 1.25) | 1.29(1.04, 1.59) | 1.34(1.07, 1.68) |
| NICOLA | 2,714 | Main model | 1.26 (0.91, 1.75) | 1.31 (0.86, 1.99) | 1.78 (1.13, 2.82) |
|  |  | Main Model +lifestyle factor | 1.25 (0.9, 1.73) | 1.28 (0.84, 1.95) | 1.76 (1.11, 2.79) |
|  | 2,700 | Main model | 1.26 (0.9, 1.75) | 1.30 (0.85, 1.99) | 1.75 (1.1, 2.79) |
|  |  | Main Model +additional indSES | 1.17 (0.84, 1.62) | 1.13 (0.74, 1.72) | 1.38 (0.86, 2.2) |
|  | 2,725 | Main model | 1.27 (0.91, 1.76) | 1.3 (0.86, 1.98) | 1.79 (1.13, 2.84) |
|  |  | Main Model +season | 1.26 (0.91, 1.76) | 1.3 (0.86, 1.98) | 1.78 (1.12, 2.82) |

Associations were further adjusted fully-adjusted models for lifestyle factors (smoking status, alcohol drinking habit, physical activity levels), additional SES (having partner, working or retirement status), or season.

Fully adjusted model included adjustment of sage, sex, calendar time, birth cohort (in HRS), race/ethnicity (except TILDA and NICOLA), individual socioeconomic status, including educational attainment at baseline, wealth, home ownership at baseline (except NICOLA), neighborhood-level socioeconomic status, urbanicity. For HRS, spatial spline term was included in the fully-adjusted model.

**Reference**

1. Sonnega A, Faul JD, Ofstedal MB, Langa KM, Phillips JW, Weir DR. Cohort Profile: the Health and Retirement Study (HRS). *Int J Epidemiol*. 2014;43(2):576-585. doi:10.1093/ije/dyu067

2. Steptoe A, Breeze E, Banks J, Nazroo J. Cohort Profile: The English Longitudinal Study of Ageing. *Int J Epidemiol*. 2013;42(6):1640-1648. doi:10.1093/ije/dys168

3. Donoghue OA, McGarrigle CA, Foley M, Fagan A, Meaney J, Kenny RA. Cohort Profile Update: The Irish Longitudinal Study on Ageing (TILDA). *Int J Epidemiol*. 2018;47(5):1398-1398l. doi:10.1093/ije/dyy163

4. Neville C, Burns F, Cruise S, et al. Cohort Profile: The Northern Ireland Cohort for the Longitudinal Study of Ageing (NICOLA). *Int J Epidemiol*. 2023;52(4):e211-e221. doi:10.1093/ije/dyad026

5. Shaddick G, Thomas ML, Green A, et al. Data Integration Model for Air Quality: A Hierarchical Approach to the Global Estimation of Exposures to Ambient Air Pollution. *J R Stat Soc Ser C Appl Stat*. 2018;67(1):231-253. doi:10.1111/rssc.12227

6. van Donkelaar A, Hammer MS, Bindle L, et al. Monthly Global Estimates of Fine Particulate Matter and Their Uncertainty. *Environ Sci Technol*. 2021;55(22):15287-15300. doi:10.1021/acs.est.1c05309

7. Larkin A, Anenberg S, Goldberg DL, Mohegh A, Brauer M, Hystad P. A global spatial-temporal land use regression model for nitrogen dioxide air pollution. *Front Environ Sci*. 2023;11:1125979. doi:10.3389/fenvs.2023.1125979

8. Becker JS, DeLang MN, Chang KL, et al. Using Regionalized Air Quality Model Performance and Bayesian Maximum Entropy data fusion to map global surface ozone concentration. *Elem Sci Anth*. 2023;11(1):00025. doi:10.1525/elementa.2022.00025

9. Malashock DA, DeLang MN, Becker JS, et al. Estimates of ozone concentrations and attributable mortality in urban, peri-urban and rural areas worldwide in 2019. *Environ Res Lett*. 2022;17(5):054023. doi:10.1088/1748-9326/ac66f3

10. Yang Y, Diez-Roux AV. Walking Distance by Trip Purpose and Population Subgroups. *Am J Prev Med*. 2012;43(1):11-19. doi:10.1016/j.amepre.2012.03.015
